# Discordant ADC and DWI signal evolution in hyperacute stroke: implications for early imaging interpretation

**DOI:** 10.1007/s00234-026-03910-3

**Published:** 2026-01-23

**Authors:** Anna Szőcs, Máté Magyar, Pál Maurovich-Horvat, Péter Barsi

**Affiliations:** 1 Medical Imaging Centre, Neuroradiology Department, Semmelweis University, Budapest, Hungary; 2 Medical Imaging Centre, Semmelweis University, Budapest, Hungary

**Keywords:** Hyperacute stroke, Diffusion MRI, ADC/DWI mismatch, Ultra-early stroke imaging

## Abstract

Here we report the first human observation of an imaging pattern in early hyperacute ischemic stroke, in which restricted diffusion is detectable on ADC maps before it becomes appreciable on DWI. The pattern—an ADC/DWI mismatch—was incidentally captured in a thalamic infarct during MRI obtained after a transient ischemic attack in a different vascular territory, enabling observation at an early hyperacute stage. Quantitative measurements at three time points demonstrated falling relative ADC and slower-rising relative DWI signal, aligning with thresholds for visual detectability. Although anticipated by animal data, this dissociation has not, to our knowledge, been documented in humans. Recognition of this putative earliest stage of ischemia may refine stroke diagnosis and inform MRI acquisition and interpretation protocols.

## Introduction

Diffusion-weighted MRI is a core modality in the diagnosis and characterization of acute ischemic stroke [1].

When critical hypoperfusion occurs, interruption of oxygen and glucose supply rapidly depletes ATP and shifts metabolism to anaerobic glycolysis, producing lactate, protons, and early acidosis. ATP-dependent ion pump failure disrupts ionic gradients and membrane potentials, triggering glutamate release and overstimulation of NMDA and AMPA receptors. The resulting Na⁺ and Ca²⁺ influx exacerbates osmotic and metabolic stress, leading to mitochondrial dysfunction and intracellular water accumulation [2]. These early changes reduce water diffusivity and cause restricted diffusion detectable as decreased ADC values within minutes of ischemic onset. However, the corresponding DWI hyperintensity may not be readily apparent in the early hyperacute phase [3].

DWI’s limitations in sensitivity are especially relevant in hyperacute, small-volume, or posterior circulation ischemia [4]. The term “DWI-negative stroke” is often used to describe such cases, but many reports focus on DWI findings alone, with no mention or depiction of ADC maps [5, 6]. Notably, DWI and ADC changes do not always evolve in parallel. In early hyperacute stroke—within the first hour—ADC reductions may precede DWI hyperintensity. This temporal dissociation has been demonstrated in animal models, including a canine study confirming that at b = 1000, ADC changes are more pronounced and earlier than DWI signal changes [7] and a rat model supporting this with higher temporal resolution [8].

Because DWI is inherently T2-weighted, its hyperintensity reflects both restricted diffusion and intrinsic T2 properties. In the minutes after ischemia, cytotoxic edema-related T2 prolongation may not yet meaningfully contribute to DWI signal. Moreover, for a given degree of restriction, ADC maps typically display higher contrast than DWI, as they render pure diffusion values without T2-weighted contributions. This relationship is described mathematically by the equation: S = S₀ × e^(-b × ADC), where DWI signal (S) depends on baseline signal (S₀, including T2 weighting) and the diffusion component [3].

In the setting of ultra-early stroke imaging, current evidence suggests ADC reduction may be the earliest imaging biomarker of ischemia and may precede detectable changes on conventional DWI. This phenomenon is distinct from other known DWI–ADC signal discordances such as “T2 shine-through”, “T2 washout,” or “T2 blackout” [9]. In addition, we would like to highlight the distinction from the term “DWI/ADC mismatch”, which in a recent study denoted the discrepancy between the visually defined DWI hyperintense region and the smaller area meeting a predefined ADC threshold (≤ 620 × 10⁻⁶ mm²/s) [10].

## Observations

We evaluated a 33-year-old Caucasian woman with a history of migraine (~ 1 attack/month) taking oral contraceptives. On October 31, 2020, at 10:00 AM, she experienced fluctuating diplopia, left-sided lip numbness, and dizziness while on the phone at work. She had no prior history of transient ischemic attacks or stroke. By emergency department arrival, her symptoms had resolved and did not recur. Neurological examination at 1:48 PM was normal.

To exclude an ischemic mechanism, brain MRI was performed on a Philips Ingenia 3T scanner (Koninklijke Philips N.V.) using a 32-channel head coil. The initial scan started at 2:48 PM, approximately five hours after symptom onset and one hour after admission to the emergency department. The imaging protocol included, among other sequences, axial DWI (single-shot echo-planar imaging; TR/TE = 3556/76 ms; field of view = 240 × 240 mm; voxel size = 1.87 × 1.87 × 5.00 mm; slice thickness = 5 mm; b = 0 and 1000 s/mm²; EPI factor = 63; SENSE parallel imaging; NSA = 2). ADC maps were automatically generated. Axial 2D FLAIR was obtained in the hyperacute and acute phases (TR/TE/TI = 4800/351/1650 ms; FOV = 230 × 185 mm; voxel size = 0.90 × 0.90 × 5.00 mm; slice thickness = 5 mm; NSA = 2), and with adjusted parameters during follow-up imaging (TR/TE/TI = 9000/125/2500 ms; FOV = 230 mm; matrix = 272 × 197 reconstructed to 560 × 560; voxel size ≈ 0.84 × 0.84 × 5.00 mm; slice thickness = 5 mm; flip angle = 90°; NSA = 1).

**Fig. 1:**
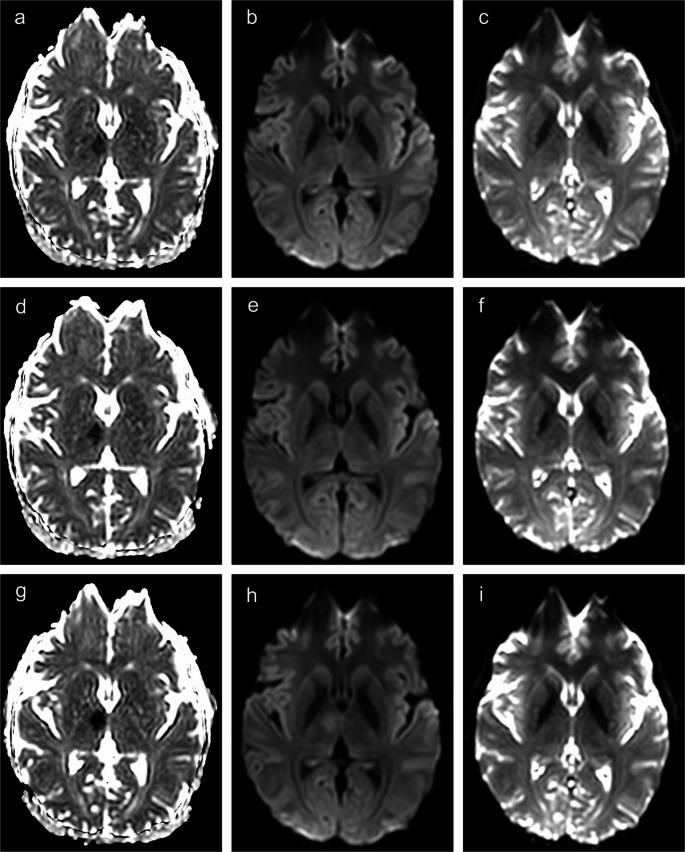
Sequential ADC (left) and DWI (middle) signal changes in a right ventromedial thalamic ischemic lesion, with corresponding b = 0 images (right). Baseline (**a**–**c**), after 6 min (**d**–**f**), and after 3.5 h (**g**–**i**)

On the initial scan (Time A), the DWI trace image did not show clearly conspicuous hyperintensity under standard clinical viewing conditions; however, the ADC map raised suspicion for a small ventromedial ischemic lesion in the right thalamus, corresponding to the territory of the mediodorsal paramedian artery originating from the P1 segment of the right posterior cerebral artery (Fig. 1a, b).

A repeat DWI sequence six minutes later (Time B), performed to verify that the ADC hypointensity did not represent motion or susceptibility artefact, showed appreciable DWI hyperintensity with a more pronounced ADC reduction (Fig. 1d, e).

A third scan was acquired 3.5 h later (Time C) to evaluate lesion evolution and assess the appearance of delayed ischemic signal change. This scan demonstrated clear changes on both sequences, with a significant signal increase on DWI and continued ADC reduction (Fig. 1g, h).

To quantify these signal dynamics, we measured DWI signal intensities and ADC values at all three time points (Table 1). Notably, while all measured relative ADC values corresponded to clear visual detection, relative DWI signal intensity values only began to be visually appreciable at approximately rSI = 1.05.

**Table 1:** Quantitative DWI signal intensity and ADC values in lesion and contralateral regions at three time points

Time	DWI-SI Lesionᵃ	DWI-SI Contralateralᵃ	rSIᵇ	ADC Lesionᶜ	ADC Contralateralᶜ	rADCᵈ
A (0′)	264	249	1.06	589	760	0.78
B (6′)	247	238	1.05	546	767	0.71
C (3.5 h)	373	264	1.41	465	776	0.58

ᵃSI = signal intensity

ᵇrSI = relative signal intensity (lesion/contralateral)

ᶜADC = apparent diffusion coefficient (×10⁻⁶ mm²/s)

^d^rADC = relative apparent diffusion coefficient (lesion/contralateral)

**Fig. 2:**
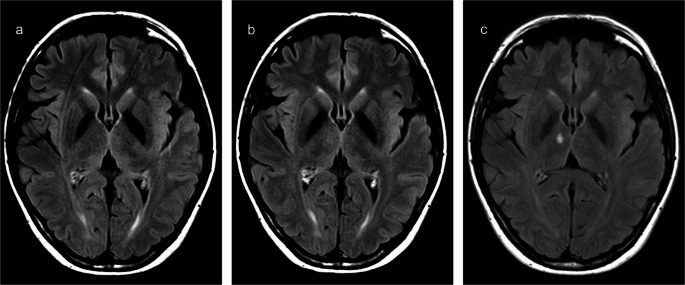
FLAIR signal changes during the hyperacute (**a**), acute (**b**), and chronic (**c**) stages of the right ventromedial thalamic stroke

A follow-up brain MRI obtained 174 days (~ 6 months) after the initial presentation showed a persistent hyperintense lesion in the same thalamic region that had exhibited the ADC/DWI mismatch, consistent with chronic post-ischemic gliosis (Fig. 2c). For comparison, FLAIR images from the early hyperacute (Time A) and acute (Time C) stages are shown alongside the follow-up scan (Fig. 2a, b), illustrating the lesion’s evolution over time.

Thrombolysis was not administered, as the patient remained asymptomatic throughout her in-hospital stay.

## Discussion

This case underscores the importance of independently evaluating DWI and ADC maps in suspected hyperacute stroke. Relying solely on DWI at this early stage could have missed the lesion, whereas the ADC provided timely diagnostic insight. Even when both are routinely reviewed, a focal ADC abnormality may be dismissed as a technical artefact if no clearly conspicuous corresponding DWI hyperintensity is present. We therefore repeated the DWI–ADC sequence six minutes later to confirm that the finding was genuine and not a technical artefact. We note that many scanners also generate e^(− b·ADC) maps, which convey information equivalent to ADC maps and may, in some cases, improve lesion conspicuity.

In the context of ultra-early stroke imaging, the observed signal discordance represents a novel and clinically relevant imaging pattern. We introduce the term *ADC/DWI mismatch* to describe an early phase of ischemic injury where ADC reduction precedes appreciable DWI hyperintensity. This may reflect the earliest stage of ischemic injury and potentially offer valuable timing information, as supported by the mentioned animal studies. The large-animal model provides valuable data but only at hourly intervals [7], whereas the rat model examined sub-hourly intervals (15, 30, and 45 min), demonstrating detectable DWI hyperintensity even at a b value of 1000 at the earliest time point [8]. Both employed anterior circulation, large-vessel occlusion paradigms. Based on these data, our case likely represents either imaging within 15 min of hypoperfusion onset, or a delayed signal response influenced by the lesion’s extent and location.

To explore whether a reduction in T2* signal might have contributed to the observed mismatch, we examined the corresponding b = 0 images at all three time points. In this context, b = 0 hypointensity could reflect an increase in local deoxyhemoglobin concentration due to elevated oxygen extraction in hypoperfused tissue. This phenomenon has been described as an early metabolic marker of ischemia that may precede cytotoxic edema, thereby potentially contributing to ADC/DWI mismatch even in the absence of hemorrhage [11]. Because ADC maps are derived from b = 0 and diffusion-weighted images, the presence of an ADC abnormality implies corresponding signal alteration on the b = 0 images, although such hypointensity may be subtle and difficult to appreciate visually, as illustrated in our case (Fig. 1c, f, i).

Taken together, our observed mismatch can primarily be explained by a combination of mechanisms. On the one hand, by the temporal evolution of ischemic injury: during the first minutes of cytotoxic edema, the degree of cellular alterations is insufficient to meaningfully increase DWI signal, whereas restricted water mobility produces an immediate and readily visible ADC decline, as detailed in the Introduction and supported by the discussed experimental data. On the other hand, lesion size and topography likely favored this pattern. Studies have shown that smaller lesions are more likely to display subtler, transient, or reversible DWI abnormalities; in one report, reversible diffusion hyperintensities were significantly smaller than persistent ones [12], suggesting different temporal dynamics than in large cortical infarcts. The thalamic location may have further contributed, as small-territory and posterior circulation strokes—such as in our case—are disproportionately represented among DWI-negative presentations [4].

In light of these considerations, we recommend that the term “DWI-negative stroke” always be interpreted in conjunction with ADC map review and, where feasible, quantitative assessment of relative ADC values. This distinction is both clinically and scientifically relevant, since some cases—like ours—exhibit a transient ADC/DWI mismatch, whereas others may represent persistent DWI negativity with or without accompanying ADC abnormalities, a distinction that warrants further clarification. Notably, many studies discussing “DWI-negative stroke” rely primarily on clinical diagnosis and report negative DWI findings without addressing corresponding ADC data [4–6].

In accordance with the discussed effects of lesion size and vascular territory, it should be emphasized that this mismatch pattern is not universal, even in confirmed ultra-early stroke imaging. For example, an early hyperacute stroke scanned 11 min post-onset showed no mismatch, likely due to larger anterior circulation involvement [13]. Some variability in this pattern may also exist among brain regions and individuals.

The single-case nature of our report limits generalization but provides valuable temporal and radiologic insight into early ischemic signal evolution. Further human observations and animal models using posterior circulation or small-territory ischemia paradigms are needed to better contextualize these findings within early hyperacute stroke imaging.

Our findings highlight ADC/DWI mismatch as an important early imaging pattern and raise questions about the sensitivity of current protocols, suggesting that future studies explore higher b-values (e.g., 2000) to improve ultra-early stroke detection.

## Data Availability

No datasets were generated or analysed during the current study.
